# A Comprehensive Review on Serum Lactate Dehydrogenase (LDH) and Uric Acid in Preeclampsia: Implications for Maternal Health and Disease Severity

**DOI:** 10.7759/cureus.56928

**Published:** 2024-03-25

**Authors:** H S Deeksha, Sandhya Pajai, Manila Reddy Eleti, Vinayak U Navalihiremath

**Affiliations:** 1 Obstetrics and Gynaecology, Jawaharlal Nehru Medical College, Datta Meghe Institute of Higher Education and Research, Wardha, IND; 2 Internal Medicine, Koppal Institute of Medical Sciences, Koppal, IND

**Keywords:** feto-maternal outcomes, pregnancy complications, biomarkers, uric acid, lactate dehydrogenase (ldh), preeclampsia

## Abstract

Preeclampsia, a hypertensive disorder unique to pregnancy, remains a significant cause of maternal and fetal morbidity and mortality worldwide. Serum lactate dehydrogenase (LDH) and uric acid have garnered attention as potential biomarkers in understanding preeclampsia's pathophysiology and clinical management. Elevated LDH and uric acid levels have been associated with disease severity and adverse outcomes, highlighting their potential utility in risk stratification and guiding management strategies. This comprehensive review explores the roles of LDH and uric acid in preeclampsia, summarizing current evidence regarding their diagnostic, prognostic, and therapeutic implications. Future research directions are also discussed, including understanding and validation studies. Integrating LDH and uric acid measurements into routine clinical practice may facilitate early detection and intervention, ultimately improving outcomes for preeclamptic pregnancies. This review underscores the importance of serum biomarkers in enhancing our understanding and managing preeclampsia, aiming to optimize maternal and fetal health.

## Introduction and background

Preeclampsia, a significant hypertensive disorder occurring during pregnancy, poses considerable risks to both maternal and fetal health globally [1]. Perinatal mortality constitutes about 15%, which includes the intra-uterine demise of the fetus (8.6%), stillbirths (2.15%), and neonatal deaths (4.3%) [1]. It is characterized by the onset of hypertension (systolic blood pressure ≥ 140 mmHg or diastolic blood pressure ≥ 90 mmHg) and proteinuria (≥ 300 mg/24 hours) after the 20^th^ week of gestation in previously normotensive women. Despite extensive research, the exact cause of preeclampsia remains elusive, underscoring the complexity of its pathophysiology [1]. Recent studies have highlighted the potential importance of specific serum biomarkers, notably lactate dehydrogenase (LDH) and uric acid, in understanding and managing preeclampsia. LDH, an enzyme involved in cellular metabolism, is elevated in various pathological conditions, including tissue ischemia, hemolysis, and complications of pregnancy, such as preeclampsia. Similarly, uric acid, a byproduct of purine metabolism, has drawn attention due to its association with endothelial dysfunction and oxidative stress, both of which are central features of preeclampsia [2].

This review aims to achieve several objectives. Firstly, it seeks to provide an overview of current knowledge regarding the pathophysiology of preeclampsia, elucidating the underlying mechanisms and contributing factors. Secondly, it aims to summarize existing literature concerning the roles of serum LDH and uric acid in the development and progression of preeclampsia, with a particular focus on their potential as diagnostic and prognostic biomarkers. Furthermore, this review aims to evaluate the clinical implications of elevated LDH and uric acid levels in preeclampsia, including their associations with disease severity and adverse feto-maternal outcomes. Finally, it intends to discuss the potential utility of LDH and uric acid measurements in guiding clinical decision-making and optimizing management strategies for patients with preeclampsia. Through addressing these objectives, this review aims to contribute to a deeper understanding of the roles played by serum LDH and uric acid in preeclampsia and their implications for feto-maternal outcomes. Ultimately, it seeks to facilitate improved risk stratification, early diagnosis, and personalized management approaches for this high-risk pregnancy complication.

## Review

Serum lactate dehydrogenase (LDH)

Overview of LDH

LDH is a crucial enzyme found in every body cell, with exceptionally high concentrations in vital organs such as the heart, liver, muscles, kidneys, lungs, and erythrocytes [3]. Its primary function lies in the anaerobic metabolic pathway and cellular respiration, facilitating the conversion of glucose into energy [3,4]. Elevated LDH levels in the serum are linked to various clinical conditions and tissue damage, rendering it a valuable marker for diagnosing and monitoring a range of diseases, including preeclampsia [3,4]. Recent research indicates a significant increase in serum LDH levels among women affected by preeclampsia, with a notable correlation observed between LDH levels and the severity of the condition [5]. Consequently, the regular monitoring of serum LDH levels can offer valuable insights into the progression and severity of preeclampsia, assisting clinicians in assessing feto-maternal outcomes [5].

Normal LDH Levels in Pregnancy

The usual range of LDH levels during pregnancy can exhibit variability. In nonpregnant adults, the reference range for LDH typically falls between 115 and 211 U/L. However, pregnancy can lead to elevated LDH levels. Research indicates that LDH levels of 600 IU/L are commonly observed in normal pregnancies, while levels exceeding this threshold (>600 IU/L) have been associated with conditions such as preeclampsia and eclampsia. Furthermore, a separate study noted that patients with mild preeclampsia exhibited a mean LDH level of 337.89±173.15 IU/l, whereas those with severe preeclampsia demonstrated a substantially higher mean LDH level of 556.41±193.02 IU/l. This finding underscores a significant correlation between serum LDH levels and the severity of preeclampsia [6-9].

Changes in LDH Levels in Preeclampsia

Serum LDH levels have emerged as a significant indicator of the severity of preeclampsia, with multiple studies demonstrating a positive correlation between elevated LDH levels and the severity of the disease. For instance, research has shown that LDH levels are notably higher in patients with severe preeclampsia compared to those with mild preeclampsia [6]. This elevation in LDH levels has been linked to a heightened incidence of maternal and fetal complications, including preterm deliveries and low birth weight infants. Additionally, other studies have observed a significant association between high LDH levels and adverse outcomes in preeclampsia patients, such as perinatal mortality and neonatal complications. Consequently, monitoring serum LDH levels is a valuable marker for assessing the severity of preeclampsia and predicting feto-maternal outcomes [9].

Mechanisms Underlying LDH Alterations in Preeclampsia

Studies have elucidated the central mechanism underlying preeclampsia, highlighting placental underperfusion, associated hypoxia, and cellular death as key factors and emphasizing LDH [10]. LDH, an intracellular enzyme facilitating the interconversion of pyruvate to lactate, is notably stimulated by hypoxia. Research findings indicate distinct alterations in LDH isoenzymes in severe preeclampsia, with LDH 2 showing a decrease, LDH 3 demonstrating elevation, and LDH 5 exhibiting significant increases in serum and placental extracts in severe cases [10]. Furthermore, LDH levels have shown a significant correlation with the severity of preeclampsia, with higher levels associated with a greater incidence of maternal and fetal complications [6,11,12]. Notably, LDH levels exceeding 800 IU/L were observed in 26% of patients with severe preeclampsia, compared to 11.5% of those with mild preeclampsia, representing a statistically significant difference [10]. Given its affordability and reliability, LDH emerges as a valuable marker for predicting the severity of preeclampsia [12]. Consequently, the mechanisms underlying LDH alterations in preeclampsia are intricately linked to hypoxia and placental cellular death, ultimately contributing to heightened LDH levels in maternal serum.

Clinical Implications of Elevated LDH in Preeclampsia

Elevated serum LDH levels carry significant clinical implications in preeclampsia, particularly concerning disease severity and feto-maternal outcomes. Consistent research findings highlight a substantial association between heightened LDH levels and the severity of preeclampsia [6,7,10,13]. Studies consistently demonstrate that LDH levels are notably elevated in women afflicted with severe preeclampsia when compared to those with milder forms of the condition, with these elevated levels correlating with increased systolic and diastolic blood pressure [6,7,10]. Furthermore, elevated LDH levels have been linked to adverse maternal and fetal outcomes, encompassing an elevated risk of maternal complications and unfavorable fetal outcomes [6,13]. Consequently, the monitoring of serum LDH levels in women with preeclampsia may hold prognostic value, aiding in the assessment of disease severity and predicting feto-maternal outcomes [13].

Potential for LDH as a Biomarker in Preeclampsia Prediction and Management

Serum LDH levels have been investigated as a potential biomarker for predicting and managing preeclampsia. Studies have consistently demonstrated significantly higher LDH levels in women diagnosed with severe preeclampsia compared to those with milder forms of the condition, with these differences being statistically significant [6,14,15]. Elevated serum LDH levels have been found to strongly correlate with the severity of the disease and adverse maternal outcomes, suggesting its potential utility as a prognostic marker for preeclampsia severity [14,15]. These findings indicate that LDH could serve as a biochemical marker to forecast adverse pregnancy outcomes in cases of preeclampsia and eclampsia, facilitating early detection and appropriate management to avert severe consequences and complications [16]. Consequently, LDH exhibits promise as a biomarker for predicting and managing preeclampsia.

Uric acid

Overview of Uric Acid Metabolism

Uric acid serves as the end product of purine metabolism in humans and certain apes, primarily synthesized in the liver, with minor synthesis occurring in the small intestine. In humans, approximately 70% of daily uric acid elimination occurs through renal excretion, with concentrations exceeding its solubility threshold, potentially leading to precipitation, particularly within joint cavities. Unlike certain other mammals, humans lack functional uricase enzyme activity responsible for uric acid breakdown, resulting in elevated uric acid levels within the body. Roughly 70% of daily uric acid production is excreted via renal pathways, while the remaining portion is eliminated through intestinal processes [17,18]. Extensive research has explored the physiological functions and pathological implications of uric acid, with investigations focusing on its associations with conditions such as hyperuricemia, metabolic syndrome, atherosclerosis, and non-alcoholic steatohepatitis. While uric acid purportedly exhibits antioxidant properties, ongoing studies scrutinize its role in inflammation and the pathogenesis of various medical conditions [19].

Normal Uric Acid Levels in Pregnancy

During the early stages of pregnancy, serum uric acid levels in non-pregnant women typically decrease, often dropping to 3 mg/dL or lower. This decline is attributed to the uricosuric effects of estrogen and the concurrent increase in renal blood flow. However, as pregnancy advances into the third trimester, uric acid levels begin to rise, reaching levels ranging from 4 to 5 mg/dL by term [20]. Reference values for uric acid in pregnancy provided by a study published on perinatology.com indicate that during the first trimester, the normal range for uric acid levels falls between 2 and 4.2 mg/dL [21]. Furthermore, another study suggests that an elevation in serum uric acid, exceeding thresholds of >240 or 300 µM/L (equivalent to 4-5 mg/dL), during early pregnancy could potentially be implicated in the subsequent development of preeclampsia [22]. Consequently, while normal uric acid levels in pregnancy may vary, they generally tend to increase as pregnancy progresses, with 4-5 mg/dL levels commonly considered normal by the time of term.

Changes in Uric Acid Levels in Preeclampsia

The role of uric acid in preeclampsia has been extensively investigated, with elevated serum uric acid levels consistently linked to the development and severity of the condition. Studies have demonstrated that uric acid levels tend to rise before the onset of preeclampsia, with the degree of elevation often correlating with the severity of the disease [20,22,23]. Uric acid has been proposed as a potential prognostic marker for preeclampsia, with some research suggesting its utility in predicting both the onset and severity of the condition [20,23]. However, the clinical utility of serum uric acid as a predictive marker remains a subject of debate, with specific studies raising questions about its reliability, particularly in distinguishing preeclampsia from gestational hypertension [20]. Despite the ongoing discussion, the association between uric acid levels and preeclampsia continues to be a focal point of research, as it is considered an essential factor in comprehending the pathogenesis and prediction of this condition.

Mechanisms Underlying Uric Acid Alterations in Preeclampsia

Increasing serum uric acid in preeclampsia is a well-established phenomenon, with multiple proposed mechanisms underlying this alteration. Research indicates that several factors contribute to increased serum uric acid levels in preeclampsia, including renal vasoconstriction, the release of fetal DNA into circulation, and placental dysfunction [24]. Moreover, uric acid has been implicated in the induction of hypertension, endothelial oxidative stress, and mitochondrial dysfunction, all of which are relevant to the pathophysiology of preeclampsia [25]. Additionally, studies have demonstrated that elevated uric acid levels are associated with hypertension, renal disease, and adverse cardiovascular events, all of which are intricately linked with the development of preeclampsia [25]. Consequently, the evidence supports the notion that uric acid may play a pathogenic role in the development of preeclampsia rather than solely serving as a marker of the disease [25].

Clinical Implications of Elevated Uric Acid in Preeclampsia

Elevated uric acid levels in preeclampsia have been extensively studied, with research suggesting its potential as a prognostic marker for the condition. Studies have consistently shown a positive association between increasing uric acid levels and the presence and severity of preeclampsia [20,23,25]. Uric acid has been implicated in endothelial dysfunction, oxidative stress, and inflammation, all of which play crucial roles in the pathogenesis of preeclampsia [22,24]. While some research has proposed uric acid as a predictor of preeclampsia, the clinical utility of using serum uric acid levels to differentiate preeclampsia from gestational hypertension has been debated, with specific studies questioning its predictive value [20].

Potential for Uric Acid as a Biomarker in Preeclampsia Prediction and Management

The potential of uric acid as a biomarker in predicting and managing preeclampsia has garnered significant attention in several studies. Uric acid has been investigated as a potential predictor of both the occurrence and severity of preeclampsia, although findings have been somewhat contradictory. Research has indicated that elevated uric acid levels are associated with increased odds of developing preeclampsia, leading to its proposal as a potential biomarker for the condition [24-26]. However, a meta-analysis has suggested that future well-designed prospective studies are necessary to deepen our understanding of uric acid's role in predicting preeclampsia [27]. Furthermore, a recent study has reported that uric acid demonstrates similar specificity to other biomarkers for diagnosing preeclampsia, albeit with potential variations in sensitivity [28].

Association between LDH, uric acid, and preeclampsia severity

Correlation Studies

The levels of serum LDH and uric acid have been identified as markers associated with the severity of preeclampsia and its implications for feto-maternal outcomes. A study revealed that LDH levels exceeding 800 IU/L were present in 26% of patients with severe preeclampsia, compared to 11.5% of those with mild preeclampsia, a statistically significant difference. Similarly, uric acid levels surpassing 8 mg/dL were associated with severe preeclampsia in 43% of cases compared to only 15.5% with mild preeclampsia, also statistically significant [2]. Another investigation found significantly elevated mean levels of LDH, uric acid, and creatinine in women with severe preeclampsia compared to those with milder forms, suggesting the prognostic value of biomarkers such as LDH, uric acid, and alanine transaminase (ALT) in detecting preeclampsia severity [6]. In a prospective case-control study, serum LDH and uric acid levels were notably higher in preeclamptic patients compared to controls, with elevated levels correlating with significant maternal and fetal complications [29]. Additionally, a separate study concluded that LDH could serve as a biochemical marker for preeclampsia severity, as its levels correlated with other parameters indicative of disease severity, unlike those of serum uric acid [5].

Predictive Value of Combined LDH and Uric Acid Levels

The combined levels of serum LDH and uric acid in preeclampsia have been investigated for their predictive value, revealing potential significance in assessing the severity of the condition and its associated outcomes. In one study, LDH levels exceeding 800 IU/L were observed in 26% of patients with severe preeclampsia, compared to 11.5% of those with mild preeclampsia, a statistically significant finding. Similarly, uric acid levels surpassing 8 mg/dL were associated with severe preeclampsia in 43% of cases compared to only 15.5% with mild preeclampsia, also statistically significant [2]. Another study reported a statistically significant increase in serum LDH and uric acid levels among preeclamptic patients compared to a control group. Elevated levels were also linked to significant maternal and fetal complications [29]. Furthermore, in a prospective case-control study, a significant increase in serum LDH and uric acid levels was noted among preeclamptic patients compared to controls, with higher levels associated with notable maternal and fetal complications [5].

Clinical Utility in Assessing Preeclampsia Severity

The clinical assessment of preeclampsia severity remains a significant challenge, prompting the exploration of practical predictive, diagnostic, and therapeutic approaches to mitigate its impact. While current clinical practices rely on risk factors and assessments such as blood pressure measurements and urinary protein analysis for triage, there is a growing interest in predictive biomarkers for preeclampsia. Research indicates that biomarkers, including serum LDH and uric acid, promise to predict the severity of preeclampsia, enabling early diagnosis, targeted surveillance, and timely intervention. For instance, a novel test has been developed with up to 96% accuracy in predicting whether patients with new-onset hypertension in pregnancy are likely to progress to severe preeclampsia [30,31]. Moreover, studies have illustrated the predictive value of biomarkers such as LDH, uric acid, and ALT in detecting preeclampsia severity, with LDH levels correlating with parameters indicative of preeclampsia severity [6].

The feto-maternal outcome in preeclampsia

Impact of Preeclampsia on Maternal Health

**Figure 1:**
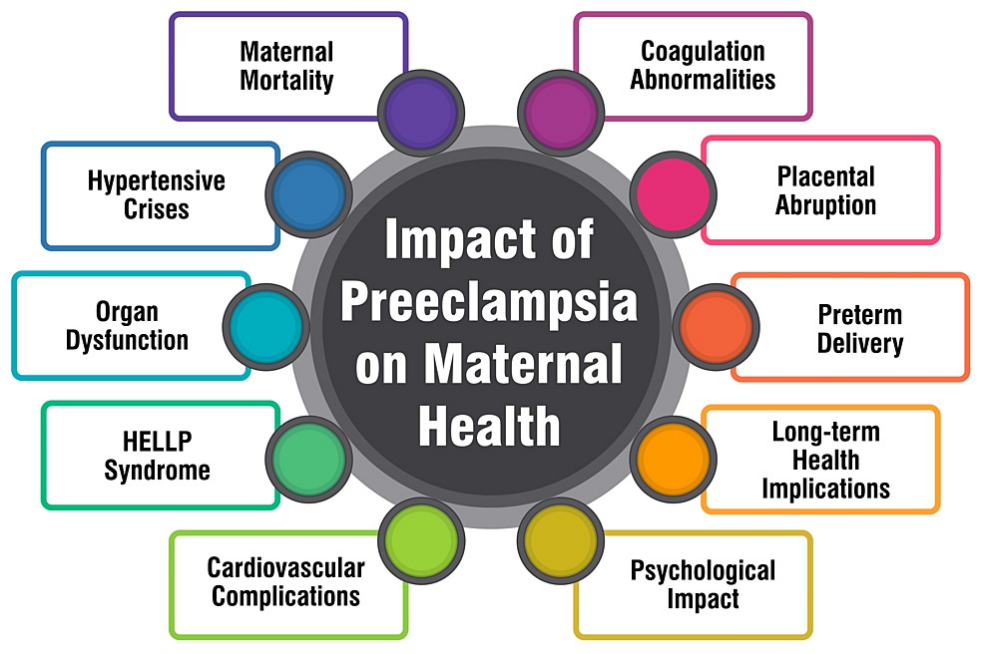
impact of preeclampsia on maternal health HELLP: Hemolysis, elevated liver enzymes, and low platelets Corresponding author H S Deeksha created this figure.

Preeclampsia represents a severe complication during pregnancy, bearing significant risks for both maternal and fetal well-being. Studies have consistently highlighted that preeclampsia with severe features, including eclampsia, can lead to substantial maternal and perinatal morbidity and mortality [32]. The severity of preeclampsia is closely linked with an elevated risk of maternal and fetal complications, encompassing outcomes such as maternal mortality, perinatal mortality, preterm delivery, low birth weight, and admission to neonatal intensive care units [32-34]. Notably, the risk of adverse outcomes is particularly heightened in cases of early-onset preeclampsia [35]. However, timely and appropriate management of the condition has been shown to mitigate adverse outcomes [34]. The impact of preeclampsia on maternal health is shown in Figure 1.

Impact of Preeclampsia on Fetal Health

**Figure 2:**
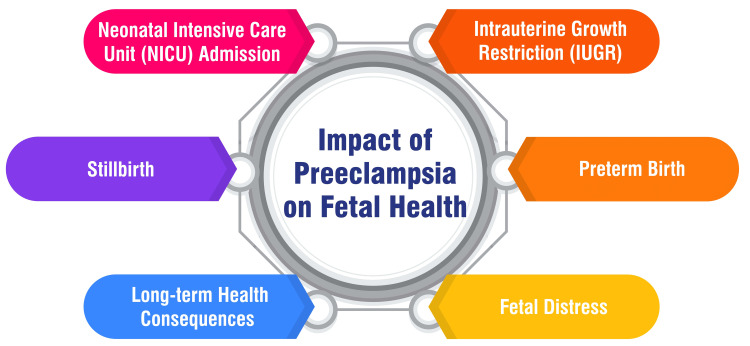
Impact of preeclampsia on fetal health Corresponding author H S Deeksha created this figure.

Preeclampsia exerts significant repercussions on fetal health, both in the short and long term. The condition is intricately linked with disturbances in fetal growth, rendering the fetus more susceptible to adverse outcomes and standing as a prominent contributor to perinatal morbidity and mortality. Severe forms of preeclampsia can precipitate varying degrees of fetal injury, primarily stemming from inadequate nutrition due to uteroplacental vascular insufficiency, ultimately resulting in growth retardation [36]. Infants born to mothers afflicted with preeclampsia face heightened risks of adverse outcomes, including impaired growth, preterm birth, stillbirth, and the onset of long-term health issues such as diabetes, congestive heart failure, and hypertension [37]. Moreover, exposure to preeclampsia in utero can leave lasting imprints on offspring, elevating their susceptibility to developing metabolic, neurological, and cardiovascular ailments later in life [38]. Additionally, preeclampsia significantly contributes to premature birth, intrauterine growth restriction, and being small for gestational age, all of which can exert enduring impacts on offspring health [38]. The impact of preeclampsia on fetal health is shown in Figure 2.

Role of LDH and Uric Acid in Predicting Adverse Outcomes

The role of serum LDH and uric acid in predicting adverse feto-maternal outcomes in preeclampsia has been extensively investigated in various studies. Findings consistently demonstrate that significant alterations in LDH and uric acid levels are associated with an increased severity of preeclampsia, with higher levels correlating with a heightened incidence of maternal and fetal complications. For example, LDH levels exceeding 800 IU/L were observed in 26% of patients with severe preeclampsia, compared to 11.5% of those with mild preeclampsia, a statistically significant difference. Similarly, uric acid levels surpassing 8 mg/dL were associated with severe preeclampsia in 43% of cases compared to only 15.5% with mild preeclampsia, also statistically significant [2]. Another study concluded that serum LDH and uric acid serve as inexpensive and reliable markers for predicting the severity of preeclampsia [29]. Additionally, research has highlighted uric acid as one of the most consistent and earliest detectable changes in preeclampsia, suggesting its potential as a superior predictor of fetal risk compared to blood pressure alone [38].

Management Strategies Based on LDH and Uric Acid Levels

**Figure 3:**
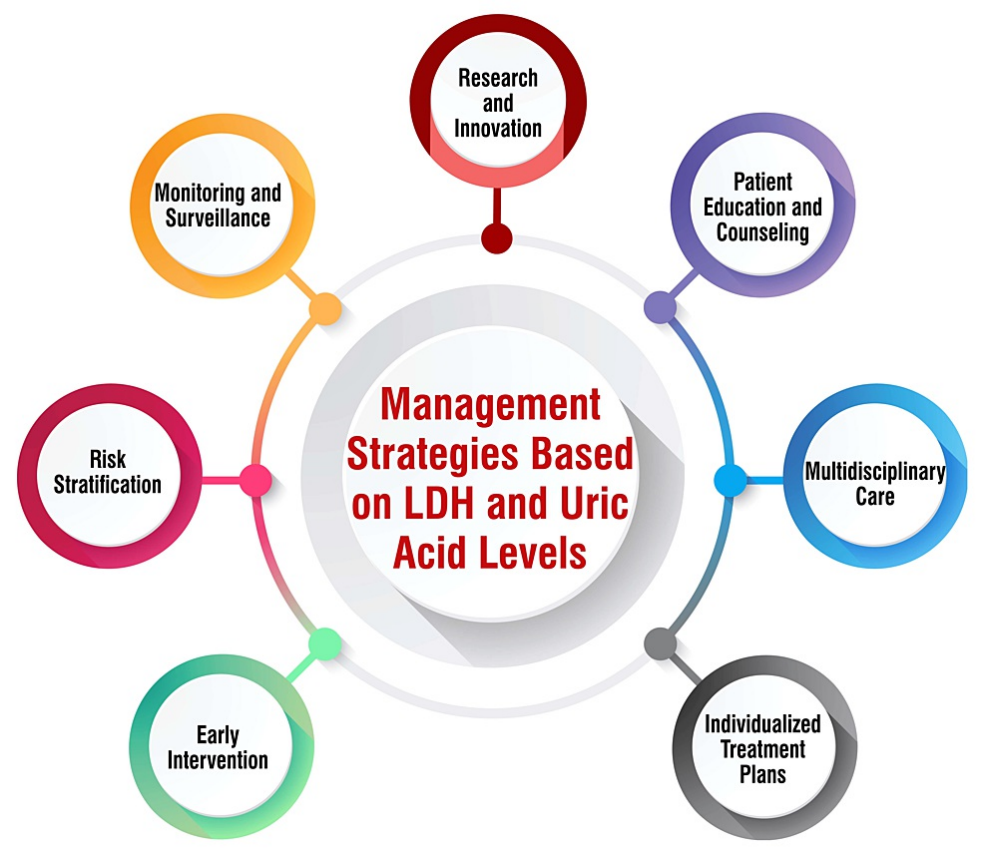
Management strategies based on LDH and uric acid levels LDH: Lactate dehydrogenase Corresponding author H S Deeksha created this figure.

Studies have underscored the utility of serum LDH and uric acid levels as predictive markers for assessing the severity of preeclampsia and its associated outcomes [2,5,28]. Consequently, monitoring these biomarkers holds promise in aiding the management of preeclampsia. For instance, research advocates for implementing standard antenatal follow-up protocols aimed at early detection and prevention of preeclampsia, particularly on rigorous monitoring of serum uric acid levels and LDH [2]. Furthermore, another study affirms that serum LDH and uric acid serve as cost-effective and dependable markers for predicting the severity of preeclampsia [29]. However, it's crucial to note that while LDH and uric acid levels offer valuable insights, no singular management strategy is solely based on these biomarkers. The management of preeclampsia is multifaceted, contingent upon factors such as the severity of the condition, gestational age, and overall maternal and fetal well-being. Hence, it is imperative to consult healthcare providers to devise and implement appropriate management strategies tailored to individual cases of preeclampsia. Management strategies based on LDH and uric acid levels are shown in Figure 3.

## Conclusions

In conclusion, the comprehensive review of LDH and uric acid levels in the context of preeclampsia offers vital insights into their pathophysiological significance and their potential as prognostic markers for assessing disease severity and feto-maternal outcomes. While the evidence underscores the intricate roles these biomarkers play in the development and progression of preeclampsia, it also highlights the complexities and challenges involved in translating this knowledge into clinical practice. The association of elevated LDH and uric acid levels with increased severity of preeclampsia and adverse outcomes underscores the need for further research to refine their predictive value and to integrate them effectively into clinical protocols. As we move forward, the pursuit of well-designed, prospective studies will be crucial in harnessing the full potential of these biomarkers, not only to improve our understanding of preeclampsia but also to enhance the management and outcomes for affected patients. Ultimately, the integration of LDH and uric acid levels into a multifaceted approach to preeclampsia care promises to advance maternal and neonatal health significantly.

## References

[1] Miller JJ, Higgins V, Ren A, Logan S, Yip PM, Fu L (2023). Advances in preeclampsia testing. Adv Clin Chem.

[2] Moharana JJ, Mishra R, Nayak AK (2023). A study on serum lactate dehydrogenase and uric acid in preeclampsia and eclampsia: can they predict adverse fetomaternal outcome?. Int J Appl Basic Med Res.

[3] (2024). Lactate dehydrogenase (LDH), serum. https://www.mayocliniclabs.com/test-catalog/overview/8344.

[4] (2024). LDH test. https://my.clevelandclinic.org/health/diagnostics/22736-lactate-dehydrogenase-ldh-test.

[5] Kunder M, Mendez DC, Shashidhar KN, Munikrishna M (2019). Correlation of serum lactate dehydrogenase and uric acid levels with severity parameters of preeclampsia. Int J Clin Biochem Res.

[6] Kasraeian M, Asadi N, Vafaei H, Zamanpour T, Shahraki HR, Bazrafshan K (2018). Evaluation of serum biomarkers for detection of preeclampsia severity in pregnant women. Pak J Med Sci.

[7] Jaiswar SP, Gupta A, Sachan R, Natu SN, Shaili M (2011). Lactic dehydrogenase: a biochemical marker for preeclampsia-eclampsia. J Obstet Gynaecol India.

[8] Reddy Eleti M, Agrawal M, Dewani D, Goyal N (2023). Serum LDH levels in normotensive and preeclamptic-eclamptic pregnant women and its correlation with fetomaternal outcome. Cureus.

[9] (2024). Reference values for lactate dehydrogenase (LDH) in pregnancy. https://www.perinatology.com/Reference/Reference%20Ranges/LDH.htm.

[10] Saleem FR, Chandru S, Biswas M (2020). Evaluation of total LDH and its isoenzymes as markers in preeclampsia. J Med Biochem.

[11] Bhati BS, Mirza N, Choudhary PK (2020). Correlation of lactate dehydrogenase levels with outcome in patients with pre-eclampsia. Adv Hum Biol.

[12] Gupta A, Bhandari N, Kharb S, Chauhan M (2019). Lactate dehydrogenase levels in preeclampsia and its correlation with maternal and perinatal outcome. Int J Reprod Contracept Obstet Gynecol.

[13] Mary VP, Chellatamizh M, Padmanaban S (2017). Role of serum LDH in preeclampsia as a prognostic factor - a cross sectional case control study in tertiary care hospital. Int J Reprod Contracept Obstet Gynecol.

[14] Mehta M, Parashar M, Kumar R (2019). Serum lactate dehydrogenase: a prognostic factor in pre-eclampsia. Int J Reprod Contracept Obstet Gynecol.

[15] Khidri FF, Shaikh F, Khowaja IU, Riaz H (2020). Role of lactate dehydrogenase in the prediction of severity in pre-eclampsia. Curr Hypertens Rev.

[16] Dave A, Maru L, Jain A (2016). LDH (lactate dehydrogenase): a biochemical marker for the prediction of adverse outcomes in pre-eclampsia and eclampsia. J Obstet Gynaecol India.

[17] El Ridi R, Tallima H (2017). Physiological functions and pathogenic potential of uric acid: a review. J Adv Res.

[18] (2024). Hyperuricemia: practice essentials, pathophysiology, etiology. Published Online First: 25 January.

[19] Kushiyama A, Nakatsu Y, Matsunaga Y (2016). Role of uric acid metabolism-related inflammation in the pathogenesis of metabolic syndrome components such as atherosclerosis and nonalcoholic steatohepatitis. Mediators Inflamm.

[20] Johnson RJ, Kanbay M, Kang DH, Sánchez-Lozada LG, Feig D (2011). Uric acid: a clinically useful marker to distinguish preeclampsia from gestational hypertension. Hypertension.

[21] (2024). Reference values for uric acid in pregnancy. https://www.perinatology.com/Reference/Reference%20Ranges/Uric%20acid.htm.

[22] Nakagawa T, Kang DH, Johnson RJ (2023). An elevation in serum uric acid precedes the development of preeclampsia. Hypertens Res.

[23] Corominas AI, Medina Y, Balconi S, Casale R, Farina M, Martínez N, Damiano AE (2021). Assessing the role of uric acid as a predictor of preeclampsia. Front Physiol.

[24] Bainbridge SA, Roberts JM (2008). Uric acid as a pathogenic factor in preeclampsia. Placenta.

[25] Colmenares-Mejia CC, Quintero-Lesmes DC, Bautista-Niño PK (2023). Uric acid and risk of pre-eclampsia: results from a large case-control study and meta-analysis of prospective studies. Sci Rep.

[26] Bellos I, Pergialiotis V, Loutradis D, Daskalakis G (2020). The prognostic role of serum uric acid levels in preeclampsia: a meta-analysis. J Clin Hypertens (Greenwich).

[27] Garrido-Giménez C, Cruz-Lemini M, Álvarez FV (2023). Predictive model for preeclampsia combining sFlt-1, PlGF, NT-proBNP, and uric acid as biomarkers. J Clin Med.

[28] Shivamurthy G, Smanjunath N (2020). Study of estimation of serum LDH and uric acid in preeclampsia and it’s clinical correlation. Int J Reprod Contracept Obstet Gynecol.

[29] MacDonald TM, Walker SP, Hannan NJ, Tong S, Kaitu'u-Lino TJ (2022). Clinical tools and biomarkers to predict preeclampsia. EBioMedicine.

[30] (2024). Clinical experience will shed more light on new preeclampsia test. https://consultqd.clevelandclinic.org/clinical-experience-will-shed-more-light-on-new-preeclampsia-test.

[31] Ajah LO, Ozonu NC, Ezeonu PO, Lawani LO, Obuna JA, Onwe EO (2016). The feto-maternal outcome of preeclampsia with severe features and eclampsia in Abakaliki, South-East Nigeria. J Clin Diagn Res.

[32] Mousa A, Mandili RL, Aljahdali M (2022). Maternal and fetal outcomes of preeclampsia with and without severe features in King Abdulaziz University Hospital, Jeddah, Saudi Arabia: a retrospective study. Cureus.

[33] Aabidha PM, Cherian AG, Paul E, Helan J (2015). Maternal and fetal outcome in pre-eclampsia in a secondary care hospital in South India. J Family Med Prim Care.

[34] Belay Tolu L, Yigezu E, Urgie T, Feyissa GT (2020). Maternal and perinatal outcome of preeclampsia without severe feature among pregnant women managed at a tertiary referral hospital in urban Ethiopia. PLoS One.

[35] (2024). Effects of preeclampsia on the mother, fetus and child. https://www.contemporaryobgyn.net/view/effects-preeclampsia-mother-fetus-and-child.

[36] (2024). What are the risks of preeclampsia & eclampsia to the fetus?. https://www.nichd.nih.gov/health/topics/preeclampsia/conditioninfo/risk-fetus.

[37] Lu HQ, Hu R (2019). Lasting effects of intrauterine exposure to preeclampsia on offspring and the underlying mechanism. AJP Rep.

[38] Roberts JM, Bodnar LM, Lain KY, Hubel CA, Markovic N, Ness RB, Powers RW (2005). Uric acid is as important as proteinuria in identifying fetal risk in women with gestational hypertension. Hypertension.

